# The Terminal Immunoglobulin-Like Repeats of LigA and LigB of *Leptospira* Enhance Their Binding to Gelatin Binding Domain of Fibronectin and Host Cells

**DOI:** 10.1371/journal.pone.0011301

**Published:** 2010-06-24

**Authors:** Yi-Pin Lin, Sean P. McDonough, Yogendra Sharma, Yung-Fu Chang

**Affiliations:** 1 Department of Population Medicine and Diagnostic Sciences, College of Veterinary Medicine, Cornell University, Ithaca, New York, United States of America; 2 Department of Biomedical Science, College of Veterinary Medicine, Cornell University, Ithaca, New York, United States of America; 3 Center for Cellular and Molecular Biology, Hyderabad, India; University of Hyderabad, India

## Abstract

*Leptospira spp.* are pathogenic spirochetes that cause the zoonotic disease leptospirosis. *Leptospiral* immunoglobulin (Ig)-like protein B (LigB) contributes to the binding of *Leptospira* to extracellular matrix proteins such as fibronectin, fibrinogen, laminin, elastin, tropoelastin and collagen. A high-affinity Fn-binding region of LigB has been localized to LigBCen2, which contains the partial 11th and full 12th Ig-like repeats (LigBCen2R) and 47 amino acids of the non-repeat region (LigBCen2NR) of LigB. In this study, the gelatin binding domain of fibronectin was shown to interact with LigBCen2R (K_D_ = 1.91±0.40 µM). Not only LigBCen2R but also other Ig-like domains of Lig proteins including LigAVar7'-8, LigAVar10, LigAVar11, LigAVar12, LigAVar13, LigBCen7'-8, and LigBCen9 bind to GBD. Interestingly, a large gain in affinity was achieved through an avidity effect, with the terminal domains, 13th (LigA) or 12th (LigB) Ig-like repeat of Lig protein (LigAVar7'-13 and LigBCen7'-12) enhancing binding affinity approximately 51 and 28 fold, respectively, compared to recombinant proteins without this terminal repeat. In addition, the inhibited effect on MDCKs cells can also be promoted by Lig proteins with terminal domains, but these two domains are not required for gelatin binding domain binding and cell adhesion. Interestingly, Lig proteins with the terminal domains could form compact structures with a round shape mediated by multidomain interaction. This is the first report about the interaction of gelatin binding domain of Fn and Lig proteins and provides an example of Lig-gelatin binding domain binding mediating bacterial-host interaction.

## Introduction

Microbial Surface Components Recognizing Adhesive Matrix Molecules (MSCRAMMs) are a group of proteins located on the surface of microbes [1]. They are able to contribute to microbial adhesion by binding to extracellular matrixes (ECMs) of host cells and initiate infection [1]. Fibronectin (Fn), a 220 kDa ECM that forms a dimer by disulfide linkage, is composed of three different modules and several different domains including an N-terminal domain (NTD), a gelatin-binding domain (GBD), a cell binding domain (CBD), a heparin binding domain II, and a fibrin-binding domain II [2], [3]. Fn plays a pivotal role in bacterial-host interaction by interacting with MSCRAMMs [4]. These MSCRAMMs may bind to NTD, GBD [5]–[7] or heparin-binding domain II [8], [9].

In the past, several potential ECM binding proteins of *Leptospira* spp. have been identified; these include Lig proteins [10]–[16], LipL32 [17]–[20], *Leptospira* endostatin-like proteins (Len) [21], [22], Lsa21 [23] and TLYC [24]. Lig proteins, including LigA, LigB and LigC, contain 13, 12, and 13 Ig-like domains, respectively [25]–[27]. The N-terminal 630 amino acid residues of LigA and LigB are highly conserve, but the C-termini are variable [25]–[27]. Unlike LigA, LigB and LigC possess a non-immunoglobulin (Ig)-like region in their C-termini [26], [27]. Lig proteins also serve as vaccine candidates and diagnostic antigens [26], [28]–[32]. Lig proteins are members of MSCRAMMs due to their ability to bind fibronectin (Fn), laminin, collagen, fibrinogen, elastin, and tropoelastin of host cells [10]–[16]. Moreover, LigBCen2, which contains LigBCen2R, the partial 11^th^ and full 12^th^ Ig-like domains, and LigBCen2NR, the C-terminal 47 non-Ig-like region, binds to NTD and GBD of Fn with high affinity that is enhanced by calcium-binding [11], [16]. In a recent report, LigBCen2NR is found to be a disordered protein and is able to fold upon NTD binding [13].

In this study, LigBCen2R was found to interact with GBD, and isothermal titration calorimetry (ITC) and surface plasmon resonance (SPR) were used to monitor the binding of GBD to Fn by proteins containing different numbers of 90 amino acid Ig-like repeats of the variable region of LigA or LigB. A large gain in affinity was achieved through an avidity effect, with the terminal domains, 13^th^ or 12^th^ Ig-like repeat of LigA or LigB (LigAVar7'-13 and LigBCen7'-12) enhancing binding affinity approximately 51- and 28-fold, respectively compared to recombinant proteins without this terminal repeat. The enhanced avidity might be due to the compact structures formed in LigAVar7'-13 and LigBCen7'-12 mediated by interdomain interaction.

## Results

### GBD binds to LigBCen2R

**Figure 1:**
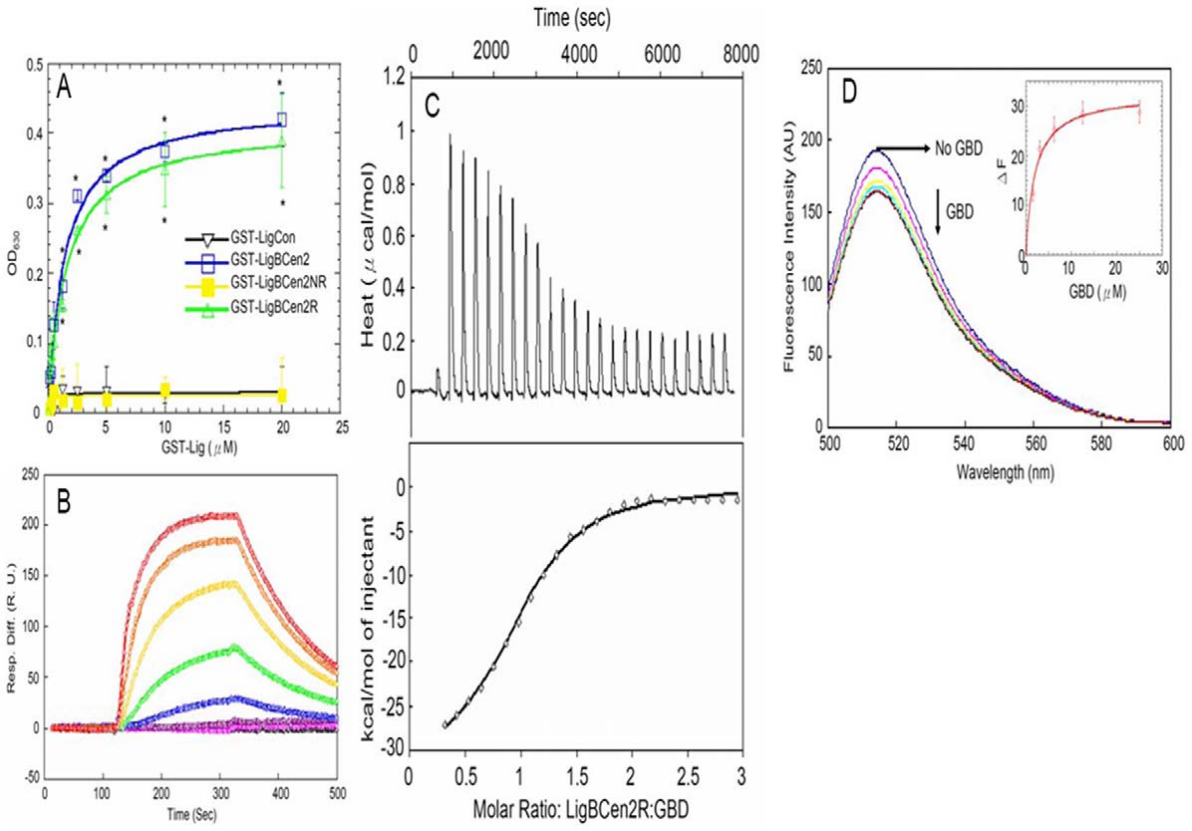
Determination of binding constant, kinetics and thermodynamic parameters of the LigBCen2R/GBD interaction by ELISA, SPR, and ITC. (A) Binding of serial concentrations of LigBCen2NR to immobilized GBD by ELISA. Serial concentrations of GST-LigBCen2R, GST-LigBCen2NR, GST-LigBCen2 (positive control), or GST-LigCon (negative control) were added to 1 µM of GBD or BSA coated wells (negative control, data not shown). (B) SPR analysis of LigBCen2R interacting with GBD. 1.5 µM of Recombinant Histidine-tagged LigBCen2R was immobilized on the surface of a Ni-NTA chip. GBD in Tris Buffer containing 100 µM CaCl2 at pH 7.5 flowed through the chip and the concentrations of GBD ranged from 40 to 0.625 µM (from top to bottom). The KD, kon, and koff were obtained from the average of duplicate experiments shown in Table 1. (C) Determination of the binding affinity by ITC. The cell contained 1 ml of GBD and the syringe contained 250 µl of LigBCen2R (upper panel). Heat differences obtained from 25 injections of LigBCen2R; (lower panel). Integrated curve with experimental data (⋄) and the best fit (-). The thermodynamic parameters are shown as the average of duplicate experiments (KD = 1.88±0.09 µM, ΔH = −29.25±3.58 kcal mol-1, TΔS = −21.47±3.61 kcal mol-1 K-1, n = 0.995±0.01). (D) Fluorescence spectrum of Alexa-488 labeled LigBCen2RW1073C in the presence and absence of GBD. One µM of Alexa-488 labeled LigBCen2RW1073C in Tris buffer was excited at 485 nm. Aliquots of GBD from respective stock solutions were added. The figure shows Alexa488 fluorescence in the presence of 0, 1.62, 3.12, 6.25, 12.5, 25 µM of GBD (Inner plot). The determination of KD of Alexa488 labeled LigBCen2RW1073C and GBD by monitoring the quenching fluorescence intensity of Alexa488 labeled LigBCen2RW1073C titrated by GBD. The emission wavelength recorded in this figure was 513 nm, and KD was revealed by fitting the data point into the equation described in materials and methods (KD = 1.93.4 µM).

In order to fine map the GBD binding site of LigBCen2, LigBCen2 was truncated into LigBCen2R and LigBCen2NR [13]. LigBCen2R and LigBCen2NR were then tested to determine if they bind to GBD. LigCon, a conserved region of both LigA and B, was included as a negative control since it does not bind to Fn [11], [12]. GBD could immobilize LigBCen2R, but not LigBCen2NR (Figure 1A). Moreover, ITC and SPR were also applied to measure the binding of LigBCen2R to GBD. The *K_D_* obtained from both experiments (ITC, *K_D_* = 1.88±0.09 µM; SPR, *K_D_* = 1.91±0.40 µM) agreed with the binding affinity of LigBCen2R-GBD obtained by ELISA (*K_D_* = 1.89±0.22 µM) (Figure 1B and C, Table 1).

**Table 1:** The dissociation constants and kinetic data of GBD-Lig interactions determined by ELISA and SPR.

Analyte	k_on_	k_off_	K_D_ a	K_D_ b
Masood A. Khan	M^−1^S^−1^×10^3^	S^−1^×10^−3^	M^−1^×10^−6^	M^−1^×10^−6^
LigAVar7'-8	0.75±0.05	5.01±0.82	6.68±0.80	6.75±0.10
LigAVar9	n.d.^c^	n.d.^c^	n.d.^c^	n.b.^d^
LigAVar10	1.19±0.43	5.06±0.25	4.25±0.72	3.94±0.32
LigAVar11	1.19±0.49	5.99±0.13	5.04±0.32	4.70±0.24
LigAVar12	2.36±0.38	6.78±0.84	2.87±0.30	2.44±0.87
LigAVar13	0.51±0.09	5.30±0.43	10.39±2.53	10.96±0.41
LigBCen7'-8	3.57±0.25	9.63±0.18	2.69±0.70	2.27±0.15
LigBCen9	0.56±0.03	4.73±0.24	8.44±0.81	8.37±0.72
LigBCen10	n.d.^c^	n.d.^c^	n.d.^c^	n.b.^d^
LigBCen11	n.d.^c^	n.d.^c^	n.d.^c^	n.b.^d^
LigBCen2R	4.83±0.54	9.23±0.24	1.91±0.40	1.89±0.22

a determined by SPR.

b determined by ELISA.

c not determined.

d no binding.

The association and dissociation data of the interaction were fitted locally using one step biomolecular reaction model (1∶1 Langmuir model : A+B
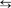
AB), which resulted optimum mathematical fits reflected by the lowest Chi values. The values for association rate constant (k_on_), dissociation rate constant (k_off_), and dissociation constant (K_D_) were calculated from the binding data by BIAevaluation software.

Significant quenching (approximately16% decrease) was found in the Alexa-488 fluorescence spectra of Alexa-488 labeled LigBCen2RW1073C, a LigBCen2 mutant lacking its sole tryptophan, when GBD was added with dose dependence confirming that GBD binding induces conformational changes in LigBCen2R (Figure 1D). The undistinguished far-UV CD spectra of LigBCen2R and LigBCen2RW1073C (Figure S1) and the close *K_D_* of the interaction of GBD-LigBCen2R and GBD-LigBCen2RW1073C determined by fluorescence spectroscopy (*K_D_* = 1.93±0.4 µM) rule out the possibility that the mutation alters the structure of LigBCen2R or the binding of LigBCen2R and GBD.

### Determination that Ig-like domains interact with GBD

**Figure 2:**
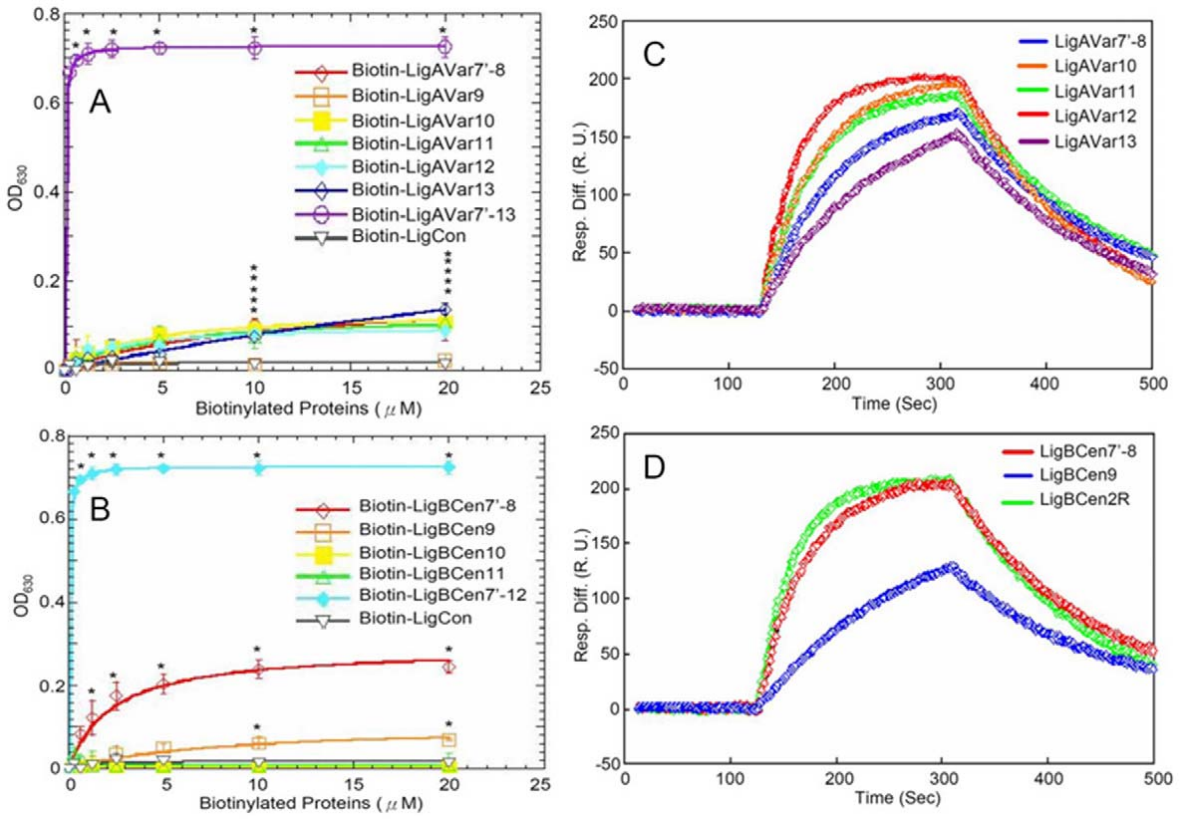
Localization of the GBD-binding domains on Lig proteins. (A and B) Various concentrations (0, 0.3125, 0.625, 1.25, 2.5, 5, 10, 20 µM) of biotinylated LigCon (negative control), and (A) LigAVar7'-13 (positive control), LigAVar7'-8, LigAVar9, LigAVar10, LigAVar11, LigAVar12, LigAVar13, (B) LigBCen7'-12 (positive control), LigBCen7'-8, LigBCen9, LigBCen10, LigBCen11 were added to wells coated with 1 µM of GBD or BSA (negative control and data not shown) in Tris buffer. The binding of biotinylated proteins to GBD was measured by ELISA. For all experiments, each value represents the mean±SEM of three trials in triplicate samples. Statistically significant (p<0.05) differences compared to the negative control are indicated by an asterisk. (C and D) SPR analysis of Ig-like domains of Lig interacting with GBD 1.5 µM of Recombinant Histdine-tag (C) LigAVar7'-8, LigAVar10, LigAVar11, LigAVar12, LigAVar13, (D) LigBCen7'-8, LigBCen9, or LigBCen2R was immobilized on the surface of Ni-NTA chip. GBD in Tris Buffer containing 100 µM CaCl2 at pH 7.5 was flowed through the chip and the concentration of GBD ranged from 40 to 0.625 µM. Only the response sensograms of the 40 µM of GBD are shown. The KD, kon, koff were obtained from the average of duplicate experiments shown in Table 1.

Since LigBCen2R containing the partial 11^th^ and full 12^th^ Ig-like domain of LigB shows GBD binding activity, we used ELISA to investigate whether the variable regions of LigA and LigB interact with GBD. As presented in Figure 2A and B, LigAVar7'-8, LigAVar10, LigAVar11, LigAVar12, and LigAVar13 in LigA and LigBCen7'-8 and LigBCen9 of LigB are able to bind GBD (LigAVar7'-8, *K_D_* = 6.75±0.10 µM; LigAVar10, *K_D_* = 3.94±0.32 µM; LigAVar11, *K_D_* = 4.70±0.24 µM; LigAVar12, *K_D_* = 2.44±0.87 µM, LigAVar13, *K_D_* = 10.96±0.41 µM; LigBCen7'-8, *K_D_* = 2.27±0.15 µM; LigBCen9, *K_D_* = 8.37±0.72 µM) (Table 1). Furthermore, a similar *K_D_* obtained from SPR also confirms the binding of certain variable regions of LigA and LigB with GBD (LigAVar7'-8, *K_D_* = 6.68±0.80 µM; LigAVar10, *K_D_* = 4.25±0.72 µM; LigAVar11, *K_D_* = 5.04±0.32 µM; LigAVar12, *K_D_* = 2.87±0.30 µM, LigAVar13, *K_D_* = 10.39±2.53 µM; LigBCen7'-8, *K_D_* = 2.69±0.70 µM; LigBCen9, *K_D_* = 8.44±0.81 µM) (Figure 2C and D, Table 1). These results suggest that the GBD also binds to certain variable regions of LigA and LigB.

### Terminal Ig-like domains enhances the both GBD binding and cell adhesion of Lig proteins

**Figure 3:**
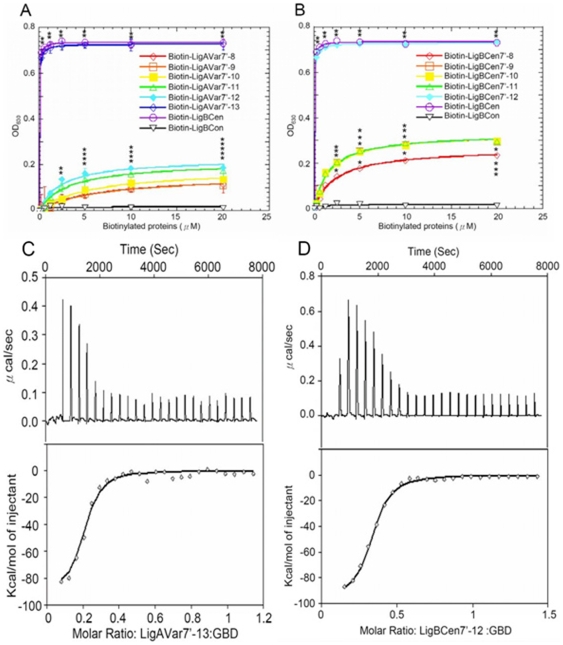
Terminal Ig-like domains mainly contributes to Lig proteins binding to GBD of Fn. (A and B) The binding of GBD to various length Lig proteins constructs performed by ELISA. Various concentrations (0, 0.3125, 0.625, 1.25, 2.5, 5, 10, 20 µM) of biotinylated LigCon (negative control), LigBCen (positive control), and (A) LigAVar7'-8, LigAVar7'-9, LigAVar7'-10, LigAVar7'-11, LigAVar7'-12, or LigAVar7'-13, (B) LigBCen7'-8, LigBCen7'-9, LigBCen7'-10, LigBCen7'-11, or LigBCen7'-12 were added to wells coated with 1 µM of GBD or BSA (negative control and data not shown) in Tris buffer. The binding of biotinylated proteins to GBD was measured by ELISA. For all experiments, each value represents the mean±SEM of three trials in triplicate samples. Statistically significant (p<0.05) differences compared to the negative control are indicated by an asterisk. The KD were obtained from the average of triplicate experiments shown in Table 2. (C and D) Representative ITC isotherms showing the binding of GBD to various length LigA or LigB constructs. The cell contained 1 ml of GBD and the syringe contained 250 µl of (A) LigAVar7'-13, and (B) LigBCen7'-12, (upper panel). Heat differences obtained from 25 injections of LigAVar7'-13 or LigBCen7'-12 (lower panel); Integrated curve with experimental data (⋄) and the best fit (-). The thermodynamic parameters are shown as the average of duplicate experiments in Table 3.

In order to investigate the effect of the number of Ig-like domains of Lig proteins on GBD binding, the truncated proteins of each construct of the variable regions of LigA and LigB were purified. The binding affinities of those truncated proteins and GBD were measured by ELISA. As shown on Figure 3A and B, a slight increase in affinity toward GBD was observed as the number of Ig-like domains increased. (LigAVar7'-8, *K_D_* = 6.75±0.10µM; LigAVar7'-9, *K_D_* = 6.58±0.40 µM; LigAVar7'-10, *K_D_* = 4.72±0.46 µM; LigAVar7'-11, *K_D_* = 3.24±0.35 µM; LigAVar7'-12, *K_D_* = 2.44±0.39 µM; LigBCen7'-8, *K_D_* = 2.27±0.15 µM; LigBCen7'-9, *K_D_* = 1.39±0.20 µM; LigBCen7'-10, *K_D_* = 1.46±0.16 µM; LigBCen7'-11, *K_D_* = 1.47±0.08 µM) (Table 2).

**Table 2:** The dissociation constants of GBD and truncated Lig proteins interactions determined by ELISA.

	K_D_		K_D_
	µM		µM
LigAVar7'-8	6.75±0.10	LigBCen7'-8	2.27±0.15
LigAVar7'-9	6.58±0.40	LigBCen7'-9	1.39±0.20
LigAVar7'-10	4.72±0.46	LigBCen7'-10	1.46±0.16
LigAVar7'-11	3.24±0.35	LigBCen7'-11	1.47±0.08
LigAVar7'-12	2.44±0.39	LigBCen7'-12	0.049±0.007
LigAVar7'-13	0.048±0.007		

**Figure 4:**
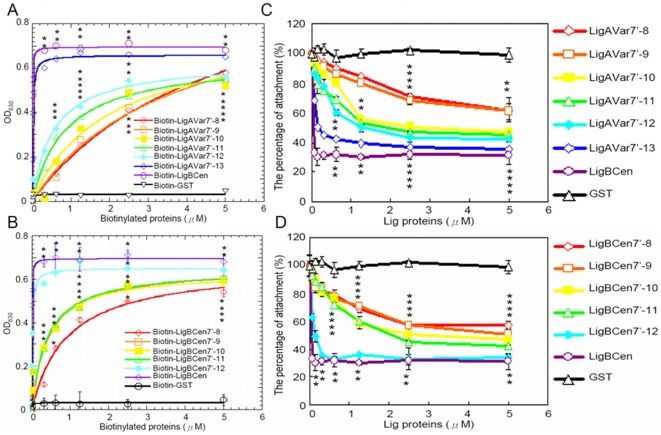
Terminal Ig-like domains mainly mediates Lig protein binding and Leptospiral adhesion on MDCK cells. (A and B) Binding of truncated Lig proteins to MDCK cells. Various concentrations (0.08, 0.16, 0.3125, 0.625, 1.25, 2.5, 5 µM) of biotinylated LigCon (negative control), LigBCen (positive control), and (A) LigAVar7'-13, LigAVar7'-12, LigAVar7'-11, LigAVar7'-10, LigAVar7'-9, LigAVar7'-8, (B) LigBCen7'-12, LigBCen7'-11, LigBCen7'-10, LigBCen7'-9, LigBCen7'-8 were added to MDCK cells (105), and binding was measured by ELISA. (C and D) Truncated Lig proteins inhibit the binding of Leptospira to MDCK cells. MDCK cells were incubated with various concentrations (0.08, 0.16, 0.3125, 0.625, 1.25, 2.5, 5 µM) of biotinylated -LigB1706-1716, or biotin (negative control) (C) LigAVar7'-13, LigAVar7'-12, LigAVar7'-11, LigAVar7'-10, LigAVar7'-9, LigAVar7'-8, (D) LigBCen7'-12, LigBCen7'-11, LigBCen7'-10, LigBCen7'-9, LigBCen7'-8 prior to the addition of Leptospira (107). The adhesion of Leptospira to MDCK cells (105) was detected by ELISA. The reduced percentage of attachment was determined relative to the attachment of Leptospira on untreated MDCK cells. Each value represents the mean±SEM of three trials in triplicate samples. Statistically significant values (P<0.05) are indicted by *.

The enhanced affinities and the stoichiometry of bindings due to the increased number of Ig-like domains of Lig proteins can also be confirmed by ITC and SECMALLS (LigAVar7'-8, *K_D_* = 6.75±0.11 µM; LigAVar7'-9, *K_D_* = 6.11±0.12 µM; LigAVar7'-10, *K_D_* = 4.35±0.43 µM; LigAVar7'-11, *K_D_* = 3.13±0.48 µM; LigAVar7'-12, *K_D_* = 2.41±0.29 µM; LigBCen7'-8, *K_D_* = 2.28±0.09 µM; LigBCen7'-9, *K_D_* = 1.31±0.19 µM; LigBCen7'-10, *K_D_* = 1.35±0.19 µM; LigBCen7'-11, *K_D_* = 1.39±0.08 µM) (Table 3 and 4 and Figure S2). Interestingly, the last Ig-like domain of both LigA and LigB contributed more than the other Ig-like domains because the GBD binding affinity of LigAVar7'-13 and LigBCen7'-12 increased 51 fold and 28 fold, respectively compared to that of LigAVar7'-12 and LigBCen7'-11, the truncated proteins lacking the last Ig-like domain (LigAVar7'-13, *K_D_* = 0.047±0.03 µM from ITC, *K_D_* = 0.048±0.007 µM from ELISA; LigBCen7'-12, *K_D_* = 0.049±0.005 µM from ITC, *K_D_* = 0.049±0.007 µM from ELISA)(Table 2 and 3). This suggests the pivotal role of 13^th^ Ig-like domain of LigA and 12^th^ Ig-like domain of LigB for the binding of Lig proteins to GBD. In order to show the physiological relevance of the importance for the terminal Ig-like domains contributing to GBD binding, the interaction of truncated Lig proteins and MDCK cells was detected by ELISA. As shown in Figure 4A and B, the more Ig-like domains included in the Lig constructs, the greater the binding of Lig proteins to MDCK cells. Furthermore, the terminal domains of Lig proteins were also proved to be pivotal since the binding was strongly promoted when the Lig constructs contained the terminal Ig-like domains (Figure 4A and B). Similarly, the inhibition of leptospiral adhesion to MDCK cells was also mediated by multivalent binding between Ig-like domains and cells due to the more significant inhibition when the cells were pre-mixed with the truncated Lig proteins including more Ig-like domains (Figure 4C and D). The ability of Lig proteins to inhibit binding was also significantly increased when the constructs included the last Ig-like domains (Figure 4C and D).

**Table 3:** Thermodynamic parameters for the interaction of GBD and truncated Lig proteins.

	[Lig Proteins]	[GBD]	ΔH	TΔS	K_D_	ΔG	n	1/n
	µM	µM	kcal mol^−1^	kcal mol^−1^ K^−1^	µM	kcal mol^−1^	Lig: GBD	GBD: Lig
LigAVar7'-8	20	1	−28.65±5.14	−21.62	6.75±0.11	−7.02	1.010±0.04	0.99
LigAVar7'-9	20	1	−29.24±4.75	−22.15	6.11±0.12	−7.08	1.020±0.08	0.98
LigAVar7'-10	20	1	−25.32±2.27	−18.04	4.35±0.43	−7.28	0.510±0.02	1.96
LigAVar7'-11	10	1	−22.08±3.31	−14.60	3.13±0.48	−7.47	0.335±0.05	2.98
LigAVar7'-12	10	2	−30.79±1.74	−23.16	2.41±0.29	−7.63	0.255±0.09	3.92
LigAVar7'-13	10	2.5	−94.89±8.0	−84.94	0.047±0.03	−9.94	0.203±0.04	4.92
LigBCen7'-8	20	1	−20.37±3.62	−12.71	2.28±0.09	−7.66	0.985±0.03	1.01
LigBCen7'-9	20	1	−21.05±5.02	−13.06	1.31±0.19	−7.99	0.520±0.05	1.92
LigBCen7'-10	20	1	−26.72±1.92	−18.75	1.35±0.19	−7.97	0.510±0.04	1.96
LigBCen7'-11	20	1	−29.01±4.23	−21.06	1.39±0.08	−7.95	0.479±0.08	2.08
LigBCen7'-12	20	4	−95.69±3.85	−85.76	0.049±0.002	−9.93	0.330±0.02	3.03

**Table 4:** Summary of Molar Ratio determined by SECMALLS.

	Calculated Molecular Weight	Binding complex	Measured molecular weight	Molar Ratio
	Da		Da	GBD: Lig
LigAVar7'-8	15,086.00	LigAVar7'-8 with GBD	56,980	0.95
LigAVar7'-9	24,572.37	LigAVar7'-9 with GBD	67,560	0.97
LigAVar7'-10	34,098.02	LigAVar7'-10 with GBD	122,800	2.00
LigAVar7'-11	43,558.46	LigAVar7'-11 with GBD	174,900	2.96
LigAVar7'-12	53,671.67	LigAVar7'-12 with GBD	232,500	4.04
LigAVar7'-13	63,015.21	LigAVar7'-13 with GBD	286,900	5.05
LigBCen7'-8	15,194.18	LigBCen7'-8 with GBD	60,070	1.01
LigBCen7'-9	24,668.05	LigBCen7'-9 with GBD	115,620	2.05
LigBCen7'-10	34,162.72	LigBCen7'-10 with GBD	123,000	2.00
LigBCen7'-11	43,789.29	LigBCen7'-11 with GBD	131,540	1.98
LigBCen7'-12	52,969.47	LigBCen7'-12 with GBD	187,300	3.03
GBD	44,308^a^			

a Reported previously [50].

### The terminal Ig-like domains contribute to compact structures of Lig proteins

LigAVar13 and LigBCen12, the terminal Ig-like domains of Lig proteins, play a very important role in GBD binding, but the GBD binding affinity of LigAVar13 or LigBCen12 (LigAVar13, K_D_ = 10.39±2.53 µM; LigBCen2R (containing LigBCen12), K_D_ = 1.91±0.40 µM) alone was not significantly different from that of other Ig-like domains (Table 1). Therefore, the global structure of Lig proteins with the terminal domains plays an important role in the binding affinity of Lig protein and GBD. As presented in Table 5, the hydrodynamic radii (R_h_) of truncated Lig proteins and other standard globular proteins were determined by dynamic light scattering or analytical ultracentrifugation, and these values obtained from both methods were in agreement with each others.

**Table 5:** Summary of hydrodynamic parameters of truncated Lig proteins in Tris pH 7.0 determined by AUC and DLS.

	Calculated Mass	f^0^_20,w_ a	f^0^_20,w_/f^0a^	R_h_ b	R_h_ c
	Da	10^−7^ g/s		nm	nm
LigAVar7'-8	15,086.00	0.25	1.14	1.78	1.57
LigAVar7'-9	24,572.37	0.38	1.30	2.7	2.35
LigAVar7'-10	34,098.02	0.56	1.56	3.89	3.81
LigAVar7'-11	43,558.46	0.74	1.82	5.20	5.25
LigAVar7'-12	53,671.67	0.90	1.94	6.25	6.03
LigAVar7'-13	63,015.21	0.57	1.13	4.01	3.79
LigBCen7'-8	15,194.18	0.23	1.10	1.62	1.56
LigBCen7'-9	24,668.05	0.37	1.32	2.63	2.46
LigBCen7'-10	34,162.72	0.55	1.57	3.82	3.62
LigBCen7'-11	43,789.29	0.76	1.88	5.31	5.37
LigBCen7'-12	52,969.47	0.51	1.14	3.60	3.45

a Calculated using Equation 4 from AUC experiment.

b Calculated using Equation 5 after substituting f^0^_20,w_ for f_0_ AUC experiment.

c Measured from DLS experiment.

**Figure 5:**
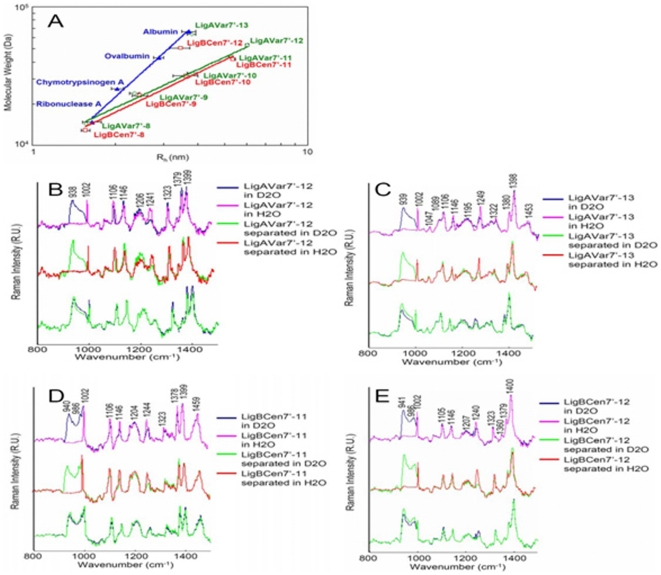
Terminal Ig-like domains contribute to the compact structure of Lig proteins. (A) Dynamic light scattering of standard molecular mass markers, LigAVar7'-13, LigAVar7'-12, LigAVar7'-11, LigAVar7'-10, LigAVar7'-9, LigAVar7'-8, LigBCen7'-12, LigBCen7'-11, LigBCen7'-10, LigBCen7'-9, LigBCen7'-8. The hydrodynamic radius (Rh) is plotted as a function of the molecular weight of each individual protein on a log-log scale. The molecular weights of the standards are indicated in Materials and Methods, and the molecular weight of the truncated Lig proteins are indicated in Table 5. (B to E) Comparative Raman spectroscopy of full-length LigAVar7'-12, LigAVar7'-13, LigBCen7'-11, LigBCen7'-12 and a stoichiometic mixture of its composite domains. The Raman spectra of full length or stoichiometrically mixed separated Ig-like domains of (B) LigAVar7'-12, (C) LigAVar7'-13, (D) LigBCen7'-11, and (E) LigBCen7'-12 were recorded in D2O and H2O.

In addition, the logarithm of R_h_ was plotted against the molecular mass of each standard using equation 3 in Materials and Methods, and a calibration curve of each globular protein or each truncated Lig protein was constructed (Figure 5A). According to the results described in Figure 5A, the R_h_ values for LigAVar7'-13 and LigBCen7'-12 are comparably closer to that of the calibration curve of the globular protein standard suggesting both LigAVar7'-13 and LigBCen7-12 are globular proteins that possess compact structures. However, the R_h_ of truncated Lig proteins without terminal Ig-like domains aligned in calibration curves distinct from the curve established by the R_h_ of the globular protein standards indicating that the Lig proteins without the terminal Ig-like domains are not globular proteins (Figure 5A and Table 5). but still possess a folded structure, especially with a rich β-strand due to the positive peak in 1241 nm and 1244 nm (amide III band) of the Raman spectra of LigAVar7'-12 and LigBCen7'-11, respectively (Figure 5B and C). Furthermore, the greater frictional ratio of the R_h_ (f^0^_20,w_/f^0^>1.2) of most truncated Lig proteins without terminal Ig-like domains also indicate the shape of them is not spherical, but the f^0^_20,w_/f^0^ of LigA7'-13 and LigB7'-12 close to 1.2 indicates the terminal Ig-like domains contribute to the compact structure of Lig proteins (Table 5) [33], [34].

In order to examine if interdomain interactions contribute to the compact structure of truncated Lig proteins with terminal domains, LigAVar7'-13, LigAVar7'-12, LigBCen7'-12, LigBCen7'-11, and stoichiometric mixture of Ig-like domains were applied to Raman spectrophotometer in H_2_O or D_2_O. The deuterium-exchange reaction showed a more significant isotopic effect on the amide III band than the amide I band. To elucidate the secondary structure of the proteins [35], the amide III band in the spectra of truncated Lig proteins were specifically identified in this study. As shown in Figure 5B, C, D, and E, the positive peaks at 1241 cm^−1^, 1249 cm^−1^, 1244 cm^−1^, 1240 cm^−1^ (amide III) observed in the spectra of full-length or stoichiometrically mixed LigAVar7'-12, LigAVar7'-13, LigBCen7'-11, and LigBCen7'-12 in H_2_O indicated that certain Lig truncations harbor a β-strand structure, which confirms the earlier findings for Ig-like domains of LigB [13], [15], [16], [35]. The positive peaks at 938 cm^−1^, 939 cm^−1^, 940 cm^−1^, 941 cm^−1^, and 986 cm^−1^ (amide III) in the spectra of Lig proteins measured in D_2_O support the conclusion that truncated Lig proteins contain a β-strand rich structure (Figure 5B, C, D, and E) [35]. Interestingly, the hydrogen-deuterium exchange (NH→ND) in full-length LigAVar7'-13 or LigBCen7'-12 was not as significant as that of stoichiometric mixture of Ig-like domains due to lower intensities of the β-strand marker, 1249 cm^−1^ and 1240 cm^−1^ in the spectrum of full-length LigAVar7'-13 or LigBCen7'-12 than stoichiometrically mixed ones (Figure 5C and E), but similar results could not be obtained withLigAVar7'-12 or LigBCen7'-11 (1241 cm^−1^ in LigAVar7'-12 1241 cm^−1^ in LigBCen7'-11) (Figure 5B and D). These results indicate that H/D exchange is more protected in full-length LigAVar7'-13 and LigBCen7'-12 compared to stoichiometrically mixed or separated Ig-like domains. However, the protection is the same between full-length LigAVar7'-12 and LigBCen7'-11 and their stoichiometric mixtures of Ig-like domains. Overall, it is proposed that more interdomain interactions exist in the truncated Lig proteins with terminal Ig-like domains, and the interdomain interactions might contribute to the compact structures of Lig proteins.

## Discussion

*Leptospira* Ig-like (Lig) proteins are MSCRAMMs that assist pathogen attachment by binding to Fn, collagen, laminin, fibrinogen, elastin, and tropoelastin [11]–[16]. LigBCen2R, containing the partial 11^th^ and full 12^th^ Ig-like domains of LigB, was demonstrated to interact with GBD of Fn. Since LigBCen2R has no sequence similarity to any other GBD binding proteins, it appears that a novel GBD binding motif is located on the Ig-like domains in the variable regions of LigA and LigB.

In addition to LigBCen2R, other variable regions of Ig-like domains of LigA and LigB were also found to possess GBD binding activity but with different binding affinity. Furthermore, the diversified *K_D_* of different variable regions of the Ig-like domains of LigA and LigB characterized by distinct association rate constants (k_on_) (5×10^−4^ to 5×10^−3^ M^−1^S^−1^) but similar dissociation rate constants (k_off_) (5×10^−3^ to 9×10^−3^ M^−1^S^−1^) suggests that there might be a conserved GBD binding motif completely or partially distributed in various variable regions of the Ig-like domains of Lig proteins. This is not surprising because the amino acid sequences of each Ig-like domain of Lig proteins are divergent [26], [27], and this inheritable difference in sequences influences not only the binding affinity to Fn but also to elastin and tropoelastin, as reported earlier [15]. Similar phenomena were also described in the interaction between a staphylococcal Fn binding protein, FnBPA, and NTD of Fn [36]. On the other hand, the comparably weaker *K_D_* and smaller k_on_ of GBD and Ig-like domains of LigA or LigB compared to other Fn binding proteins indicates a possible role of Lig proteins in transmission of *Leptospira* [37], [38], and this phenomenon was also discovered in the NTD binding region of LigB, LigBCen2NR [13].

Surprisingly, Lig proteins lacking the last Ig-like domain bound to GBD much more weakly than the constructs containing the whole variable domain, but the binding affinity between GBD and the last Ig-like domain of LigA or LigB, LigAVar13 or LigBCen2R was low. Clearly, the terminal domain of Lig protein serves an important role but is not required for GBD binding. In addition, the increased affinity of LigAVar7'-13-GBD or LigBCen7'-12-GBD could be attributed to the precipitously reduced enthalpy compared to LigAVar7'-12-GBD or LigBCen7'-11-GBD. Interestingly, the structural differences of the Lig constructs with or without the terminal domain were also revealed by DLS, Raman spectrometry, and AUC and all indicated that Lig proteins with the terminal Ig-like domains exhibit compact structures attributable to interdomain interactions. In a previous study, multiple repeat domains of Eap from *S. aureus* could form an elongated but structured conformation mediated by interdomain interaction [39]. Thus, the structure of protein-ligand interactions requires substantial cooperative interdomain interaction. It also suggests that the conformation changes of both LigAVar7'-13 and LigBCen7'-12 made them easier to access GBD resulting in a greater reduction in enthalpy due to more charge-charge interactions or hydrogen bonds formed between Lig proteins and GBD. In addition, Lig proteins with the terminal domains (LigAVar7'-13 and LigBCen7'-12) possessing a high affinity binding to GBD and MDCK cells further described that this specific compact structure of Lig proteins presented the physiological significance contributing to the *Leptospira*-host interaction. (Table 2, 3 and Figure 4).

Fibronectin serves different roles mediated by distinct domains and isoforms. Two Fn isoforms include soluble plasma Fn and insoluble cellular Fn [2], [3]. MSCRAMMs are a group of proteins that allow pathogens to either attach on host cells by binding to cellular ECMs or to be decorated by plasma Fn to evade the host immune response in the blood stream [1]. Multivalency, which promotes higher binding avidity and efficiency, has been described for some MSCRAMMs such as the FnBR of SfbI of *Streptococcus pyogenes* and FnBPA or FnBPB of *S. aureus* [36], [40], [41]. Thus, *Leptospira* Lig proteins, which possess 90 amino acid Ig-like repeated domains that bind to Fn, could be reasonably inferred to serve a similar role. Recently, a novel role for MSCRAMMs was ascribed to a 70 kDa domain that includes the NTD and GBD of Fn. Both the NTD and GBD contain IGD motifs, called migration stimulating factor (MSF) located on ^3^F1, ^5^F1, ^7^F1, and ^9^F1, which mediate fibroblast migration [42], [43]. Whether Lig proteins aid *Leptospira* spp. infection through this strategy waits to be determined.

In conclusion, we have fine mapped the GBD binding sites on LigBCen2 and found GBD binds to LigBCen2R, the partial 11^th^ and full 12^th^ Ig-like domains. Furthermore, most of the individual Ig-like domains from the variable region of Lig A and LigB were also bound by GBD but with divergent affinities. Multivalent binding was proved to mediate the GBD-Lig proteins interaction and MDCK cell adhesion, and the terminal Ig-like domain serves a role for the formation of compact and round structures and a substantial but nonessential role for the interaction. Further studies on the function of Lig proteins interacting with GBD are needed and are currently being investigated in our laboratory.

## Materials and Methods

### Bacterial strains and cell culture

*L. interrogans* serovar Pomona (NVSL1427–35–093002) was used as previously described [11]. All experiments were performed with virulent, low-passage strains obtained by passage through golden Syrian hamsters as described earlier [11]. Leptospires were grown in EMJH medium at 30°C for less than 5 passages and growth was monitored by dark-field microscopy. Madin–Darby canine kidney (MDCK) cells (ATCC CCL34) were cultured in Dulbecco minimum essential medium (DMEM) containing 10% fetal bovine serum (Gibco Laboratories, Grand Island, NY). Cells were grown at 37°C in a humidified atmosphere with 5% CO_2_.

### Reagents and antibodies

Rabbit anti-GST antibody was purchased from Molecular Probes (Eugene, OR). HRP-conjugated goat anti-rabbit antibody and HRP-conjugated streptavidin were obtained from KPL (Gaithersburg, MD). Alexa Fluor 488 C_5_ maleimide and tris-(-2-carboxyethyl) phosphine (TCEP) were purchased from Molecular Probe (Carlsbad, CA). Gelatin-binding domain of human plasma fibronectin (GBD) and bovine serum albumin (BSA), Tris-HCl, calcium chloride, sodium phosphate dehydrate, and sodium chloride were obtained from Sigma-Aldrich (St. Louis, MO). D_2_O was ordered from Cambridge Isotope Laboratories (Andover, MA).

### Plasmid construction

**Figure 6:**
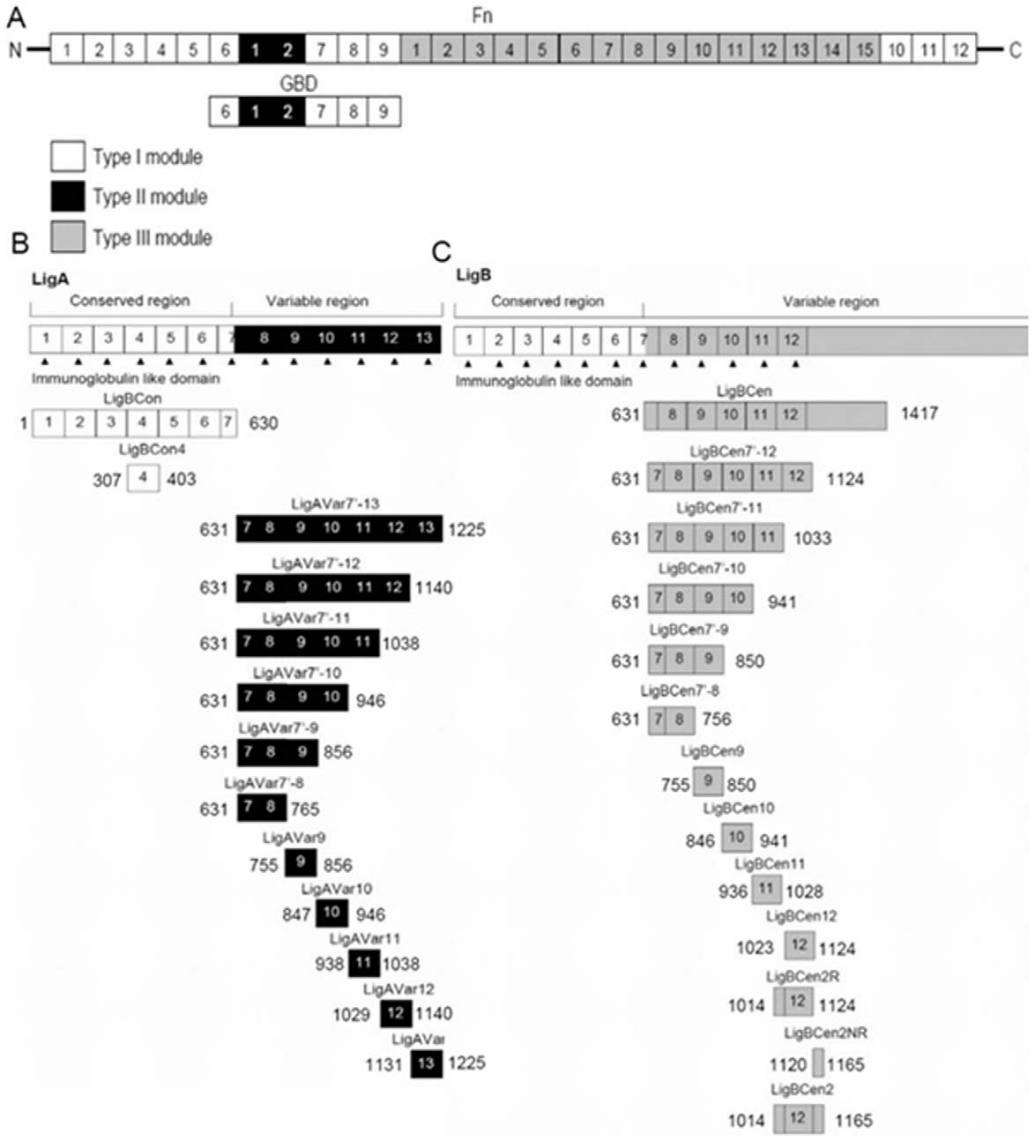
A schematic diagram showing the structure of truncated (A) Fn, (B) LigA, and (C) used in this study.

All constructs used in this study were cloned into either a pGEX-4T-2 vector (GE Healthcare, Piscataway, NJ) or a pQE-30 vector (Qiagen, Alencia, CA) and purified either as Glutathione-S-Transferase (GST) or Histidine tagged fusion proteins ( and Figure 6) [15], [16]. The concentrations of proteins were determined by the absorption in a UV spectrophotometer at 280nm, and the extinction coefficients are listed in Table S1. To make all the above constructs, PCR reactions were performed by utilizing the primers described in Table S2. The construction of LigBCon (AA 1–630), LigBCen7'-8 (AA 631–765), LigBCen9 (AA 755–850), LigBCen10 (AA 846–941), LigBCen11 (AA 936–1028), LigBCen2 (1014–1165), LigBCen2R (AA 1014–1124), LigBCen2NR (AA 1120–1165), LigAVar7'-13 (AA631–1225), LigBCen (AA631–1225) was described previously (11–13, 15, 31). For constructing LigAVar7'-8 (AA 631–765), LigAVar7'-9 (AA 631–856), LigAVar7'-10 (AA 631–946), LigAVar7-11 (AA 631–1038), LigAVar7'-12 (AA 631–1140), LigAVar9 (AA 756–856), LigAVar10 (AA 847–946), LigAVar11 (AA 938–1038), LigAVar12 (AA 1029–1140), and LigAVar13 (AA 1131–1225), primers were engineered to introduce a BamHI site at the 5′ end and a SalI site at the 3′ end of each fragment. For constructing LigBCen7'-9 (AA 631–850), LigBCen7'-10 (AA 631–941), LigBCen7'-11 (AA 631–1033), primers were engineered to introduce a BamHI site at the 5′ end and a PstI site at the 3′ end of each fragment. For constructing LigBCen7'-12 (AA 631–1124), primers were engineered to introduce a SphI site at the 5′ end and a HindIII site at the 3′ end of each fragment. PCR products were sequentially digested with BamHI and SalI, BamHI and PstI, or SphI and HindIII and then ligated into pQE30, pGEX-4T-2, or pET-THGT cut with BamHI and SalI, BamHI and PstI or, SphI and HindIII, respectively as previously described [15], [31]. For LigBCen2RW1073C mutant construction, the manufacturer's instructions of the Quickchange mutagenesis kit (Strategene, La Jolla, CA) were followed and has been described earlier [15].

### GBD binding assays by ELISA

1 µM of GBD or BSA (negative control and data not shown) in Tris buffer (25 mM Tris and 150 mM sodium chloride, pH 7.0) containing 100 µM of calcium chloride were coated onto microtiter plate wells as described previously [11]. 100 µL of different concentrations of GST fused LigBCon (negative control), LigBCen2 (positive control), LigBCen2R, or LigBCen2NR, (Figure 1A), Histidine tagged LigBCon4 (negative control), LigBCen2 (positive control), LigBCen2R (positive control), LigBCen12, or biotinylated LigAVar7'-8, LigAVar9, LigAVar10, LigAVar11, LigAVar12, LigAVar13, LigBCen7'-8, LigBCen9, LigBCen10, LigBCen11, LigBCen7'-12 (positive control), LigAVar7'-13 (positive control), (Figure 2A and B), LigAVar7'-8, LigAVar7'-9, LigAVar7'-10, LigAVar7'-11, LigAVar7'-12, LigBCen7'-8, LigBCen7'-9, LigBCen7'-10, LigBCen7'-11, LigBCen7'-12, LigBCen (positive control), (Figure 3) or LigCon (negative control) in Tris buffer containing 100 µM of calcium chloride were added subsequently. To measure the interaction of GBD and histidine tagged or GST fusion proteins, rabbit anti-GST or mouse anti-histidine tagged (1∶200) and horseradish peroxidase-conjugated goat anti-rabbit IgG or goat anti-mouse IgG (1∶1000) served as primary and secondary antibodies, respectively. To detect binding of the biotinylated proteins, HRP-conjugated streptavidin (1∶1000) was added to each well at RT for 1 hour prior to washing the wells thrice with TBST. The measurement of binding by ELISA was as described previously [15], [16]. To determine the dissociation constant (*K_D_*), the data were fitted by the following equation using KaleidaGraph software (Version 2.1.3 Abelbeck software, Reading, PA), and the calculated *K_D_* are listed in Table 1.
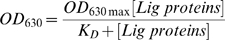
(1)


### Cell binding and inhibition assays by ELISA

To detect the binding of truncated Lig proteins to MDCK cells, MDCK cells (10^5^) were incubated with 0, 0.08, 0.16, 0.3125, 0.625, 1.25, 2.5, or 5 µM of biotinylated LigBCen (positive control), GST (negative control), or truncated Lig proteins in 100 µL PBS for 1 h at 37°C (Figure 4A and B). For measuring the binding inhibition of *Leptospira* to MDCK cells by truncated Lig proteins, MDCK (10^5^) cells were treated with 0, 0.08, 0.16, 0.3125, 0.625, 1.25, 2.5, or 5 µM of truncated Lig proteins, LigBCen (positive control), or GST (negative control) in 100 µL PBS for 1 h at 37°C prior to the addition of *Leptospira* (10^7^) for 6 h at 37°C (Figure 4C and D). To detect the binding of biotinylated Lig proteins, HRP-conjugated streptoavidine (1∶1000×) was added subsequently. To measure the binding of *Leptospira*, hamster anit-*Leptospira* (1∶200×) and HRP-conjugated goat anti-hamster IgG (1∶1000×) were used as primary and secondary antibodies, respectively. The percentage of attachment was determined relative to the attachment of serovar Pomona on untreated MDCK cells. The measurement of binding by ELISA was as described previously [15], [16]. Each value represents the mean ± SEM of three trials in triplicate samples. Statistically significant (P<0.05) differences are indicated by *.

### Surface Plasmon Resonance (SPR)

Association and dissociation rate constants for the interaction of Lig proteins and GBD were measured by SPR analysis performed with a Biacore 2000 instrument (GE Healthcare) at 25°C. 1.5 µM of each His-tagged Lig protein, including LigAVar7'-8, LigVar10, LigAVar11, LigAVar12, LigAVar13, LigBCen7'-8, LigBCen9, or LigBCen2R in Tris buffer containing 100 µM calcium chloride, was immobilized on a NTA chip (GE Healthcare) conjugated with 500 µM nickel sulfate. Serial concentrations (0, 0.625, 1.25, 2.5, 5, 10, 20, 40 µM) of GBD were injected into the flow cell at a flow rate of 5 µL/min over the immobilized Lig proteins. All experiments were repeated twice. The sensogram data were corrected by subtracting data from a control cell injected with Tris buffer containing 100 µM calcium chloride. Kinetic parameters were obtained by fitting the data to the one-step biomolecular association reaction model (1∶1 Langmuir model) with the curve-fitting BIAevaluation software, version 3.0.

### Isothermal Titration Calorimetry (ITC)

The experiments were carried out with a CSC 5300 microcalorimeter (Calorimetry Science Corp. Lindon, UT, USA) at 25°C as previously described [15]. In a typical experiment, the cell contained 1 ml of a GBD solution and the syringe contained 250 µl of a solution of LigBCen2R, LigAVar7'-8, LigAVar7'-9, LigAVar7'-10, LigAVar7'-11, LigAVar7'-12, LigAVar7'-13, LigBCen7'-8, LigBCen7'-9, LigBCen7'-10, LigBCen7'-11, or LigBCen7'-12. The concentration of LigBCen2R and GBD were 127 µM and 6 µM. The concentration of GBD and truncated Lig proteins are detailed in Table 3. Both solutions were in Tris buffer containing 100 µM of calcium chloride. The titration was performed as follows: 25 injections of 10 µl with a stirring speed of 250 rpm with a delay time of 5 min between injections. Data were analyzed using the Titration Bindworks software (Model CSC 5300, Calorimetry Science Corp. Lindon, UT, USA) fitting them to an independent binding model.

### Size exclusion chromatography Multiangle Laser Light Scattering (SECMALLS)

100–300 µg of protein was loaded in specific mixtures onto a Superose 6 column (GE Healthcare) at a flow rate of 0.5mL/min in 20mM HEPES, 150mM NaCl, pH 7.5. The column was connected to an in-line 18-angle Wyatt Down Heleos laser light-scattering detector and a Wyatt Optilab rEX refractive index detector (Wyatt Technology, Santa Barbara, CA). Molecular masses were calculated from laser light-scattering data by ASTRA V (version 5.3.0.18) software package. A refractive index increment value (dn/dc) of 0.185 ml/g was used. Detectors were normalized to compensate for slight differences in electronic gain using bovine serum albumin as an isotropic scatterer [44].

### Steady State Fluorescence Measurement

Steady state fluorescence emissions were measured on a Hitachi F7500 spectrofluorometer (Hitachi. San Jose, CA). All spectra were recorded in correct spectrum mode of the instrument using excitation and emission band passes of 2 nm. In order to measure the binding of GBD to LigBCen2R, LigBCen2RW1073C was expressed and labeled with Alexa-488. The labeling of Alexa-488 to LigBCen2RW1073C by the interaction of the cysteine of LigBCen2RW1073 with Alexa Fluor 488 C_5_ maleimide was performed following the manufacturer's instructions (Molecular Probe). For the GBD titration, 1.62, 3.12, 6.25, 12.5, 25 µM of GBD in Tris buffer containing 100 µM of calcium chloride was mixed with 1 µM of Alexa-488 labeled LigBCen2RW1073C in the same buffer. The fluorescence from Alexa-488 probe of Alexa-488 labeled LigBCen2RW1073C was recorded at the excitation wavelength of 490 nm, and the emission wavelength ranged from 500 to 600 nm. All spectra were recorded at 25°C after 5 minutes. Furthermore, the spectra of the various concentrations of GBD indicated above were also recorded and used to subtract the spectra of each Alexa-488 labeled LigBCen2RW1073C in the addition of certain concentrations of GBD. To determine the dissociation constant (*K_D_*), the fluorescence intensities at 518 nm were recorded and fitted by the following equation using KaleidaGraph software (Version 2.1.3 Abelbeck software):
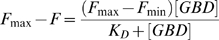
(2)Where F_max_ is the fluorescence intensity of Alexa-488 labeled LigBCen2RW1073C in the absence of GBD, and F_min_ indicates the fluorescence intensity of Alexa-488 labeled LigBCen2RW1073C saturated with GBD. In addition, F is the fluorescence intensity of Alexa-488 labeled LigBCen2RW1073C in the presence of various concentrations of GBD. All of the measurements were corrected for dilution and for inner filter effect.

### Dynamic Light Scattering (DLS)

One mg/mL of LigAVar7'-8, LigAVar7'-9, LigAVar7'-10, LigAVar7'-11, LigAVar7'-12, LigAVar7'-13, LigBVar7'-8, LigBVar7'-9, LigBVar7'-10, LigBVar7'-11, or LigBVar7'-12 was dialyzed against prefiltered (0.22 µm Millipore filters) Tris buffer with 100 µM calcium chloride. The samples were placed in a 1 mL plastic cuvette. The standard globular proteins including albumin (67,000 Da), ovalbumin (43,000 Da), chymotrypsinogen A (25,000 Da), ribonuclease A (13,700 Da), aprotinin (6,500 Da), or insulin chain B (3,400 Da) were used to generate the calibration curve of globular proteins. The automated measurements were collected with a Zetasizer Nano ZS instrument (Malvern Instruments Ltd., Worcestershire, United Kingdom), using a 2 min equilibrium delay at each measurement. The data were adjusted using the method of cumulants to obtain the hydrodynamic radius (R_h_). The logarithms of the R_h_ values of standards and above proteins were plotted against the logarithm of the protein molecular weights to fit the equation
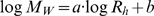
(3)where a and b are constants.

### Analytical Ultracentrifugation (AUC)

Sedimentation velocity was performed in the Beckman Optima XL-I analytical ultracentrifuge (Brea, CA) at initial loading concentration from 0.25 to 5mg/mL. 420µL aliquots of truncated Lig proteins were loaded into the sample channels of double-sector, 12nm center pieces and 410µL of Tris buffer with 100 µM calcium chloride into the corresponding reference channels. Centrifugation was carried out in an AnTi-60 rotor at 40,000 rpm for 8 hour at 20°C. Radial absorbance scans were collected in continuous scan mode at 280nm every 2 min with two replicates and a step size of 0.003 cm.

Data analysis was using SedFit and Sednterp [45], [46]. Experimental sedimentation and diffusion coefficient (S and D) were corrected to the equivalanet values in water at 20°C (S_20,w_ and D_20,w_) and then extrapolated to zero protein concentration in Sednterp to gibe the S^0^_20,w_ and D^0^_20,w_ values [45]. Sedimentation data were used to calculate the frictional coefficient f^0^_20,w_ and frictional ratio (f^0^_20,w_/f^0^) of truncated Lig proteins defined in equation 4 [47].
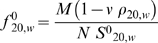
(4)Where M is molecular weight, N is Avogadro's number, ν is the partial specific volume (ml/g) of truncated Lig proteins, ρ_20,w_ is the density of the solvent (g/ml) at 20°C, and f^0^ is the frictional coefficient of a hard, unhydrated spherical particle and is defined in Equation 5 and as follows [47], and f^0^_20,w_ is f^0^ obtained at 20°C.
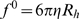
(5)Where η is the viscosity of water at 20°C and R_h_ is the radius of the sphere of the particle with molar weight of truncated Lig proteins at 20°C. Furthermore, by using values of f^0^_20,w_ calculated from Equation 5, R_h_ for sedimenting species of truncated Lig proteins was calculated using equation analogous to Equation X. The frictional ratio (f^0^_20,w_/f^0^) was calculated from f^0^_20,w_/f^0^. All results were from duplicate experiments.

### Raman Spectroscopy

LigAVar7'-12, LigAVar7'-13, LigBCen7'-11, LigBCen7'-12 or the mixture of certain Ig-like domains of LigA or LigB was concentrated with an Amincon ultrafiltration device (Millipore, Billerica, MA) to the final concentration as 3 mg/mL determined by UV absorption spectrophotometry and dialyzed against Tris buffer with 100 µM of calcium chloride [48]. For the experiments performed in D_2_O, the proteins were lyophilized, dissolved by D_2_O, and stored at 4°C for 20 hours prior to acquisition of Raman spectra. A 10 µL aliquot of the protein solution was applied to a Renishaw InVia micro-Raman spectrophotometer using long-range 50× objective, 30sec. integration, and 10% laser power (785 nm excitation; 8 mW at 100%) [49]. The spectra were corrected by subtracting the buffer and background. Peak height was normalized to 1002 cm^−1^ band of phenylalanine as an internal standard [48].

### Circular dichroism (CD) spectroscopy

CD analysis was performed on an Aviv 215 spectropolarimeter (Lakewood, NJ) under N_2_ atmosphere. CD spectra were measured at RT (25°C) in a quartz cell of appropriate path length. Spectra of 10 µM of LigBCen2R, and LigBCen2RW1073C were recorded in HEPES buffer (20mM HEPES, 150mM NaCl, pH 7.5) with 100 µM calcium chloride. Structural changes in LigBCen2R upon binding to the GBD of Fn were examined by analyzing changes in the CD spectrum. To determine the secondary structure, three far-UV CD spectra of all those proteins mentioned above were recorded from 190 to 250 nm for far-UV CD in 1 nm increments at 25°C. The background spectrum of buffer without protein was subtracted from the protein spectra. CD spectra were initially analyzed by the software accompanying the spectrophotometer. Analysis of spectra to extrapolate secondary structures was performed by Dichroweb (http://www.cryst.bbk.ac.uk/cdweb/html/home.html) using the K2D and Selcon 3 analysis programs [11], [12].

### Statistical analysis

Significant differences between samples were determined using the Student's t-test following logarithmic transformation of the data. Two-tailed P-values were determined for each sample and a P-value <0.05 was considered significant. Each data point represents the mean ± standard error of the mean (SEM) for each sample tested in triplicate. An (*) indicates the result was statistically significant.

## Supporting Information

Figure S1 W1073C mutation cannot affect the structure of LigBCen2R. Far-UV CD analysis of LigBCen2R and LigBCen2R. The molar ellipticity, Φ, was measured from 190 to 250 nm for 10 µM of each protein in Tris buffer with 100 µM of calcium chloride.
(0.94 MB TIF)

Figure S2 Representative SECMALLS analysis of the molar ratio of LigBCen7'-8-GBD complex. The data traces of SECMALLS from the instrument's three in-line detectors, measuring the refractive index, light scattering, and UV absorbance (280nm), are shown in arbitrary unit. Molecular weight was determined for the major species using the data within the shaded area. Shaded area 1, 2, and 3 indicate LigBCen7'-8-GBD complex, GBD, and LigBCen7'-8, respectively. The result of molar ratio was shown on Table 4.
(1.66 MB TIF)

Table S1 The sources of clones used in this study.
(0.06 MB DOC)

Table S2 Primer Table
(0.05 MB DOC)

## References

[1] Patti JM, Allen BL, McGavin MJ, Hook M (1994). MSCRAMM-mediated adherence of microorganisms to host tissues.. Annu Rev Microbiol.

[2] Potts JR, Campbell ID (1994). Fibronectin structure and assembly.. Curr opin cell Biol.

[3] Vakonakis I, Campbell ID (2007). Extracellular matrix: from atomic resolution to ultrastructure.. Curr Opin Cell Biol.

[4] Schwarz-Linek U, Hook M, Potts JR (2004). The molecular basis of fibronectin-mediated bacterial adherence to host cells.. Mol Microbiol.

[5] Joh D, Speziale P, Gurusiddappa S, Manor J, Hook M (1998). Multiple specificities of the staphylococcal and streptococcal fibronectin-binding microbial surface components recognizing adhesive matrix molecules.. Eur J Biochem.

[6] Joh D, Wann ER, Kreikemeyer B, Speziale P, Hook M (1999). Role of fibronectin-binding MSCRAMMs in bacterial adherence and entry into mammalian cells.. Matrix Biol.

[7] Probert WS, Kim JH, Hook M, Johnson BJ (2001). Mapping the ligand-binding region of *Borrelia burgdorferi* fibronectin-binding protein BBK32.. Infect Immun.

[8] Dabo SM, Confer AW, Anderson BE, Gupta S (2006). *Bartonella henselae* Pap31, an extracellular matrix adhesin, binds the fibronectin repeat III13 module.. Infect Immun.

[9] Kingsley RA, Keestra AM, de Zoete MR, Baumler AJ (2004). The ShdA adhesin binds to the cationic cradle of the fibronectin 13FnIII repeat module: evidence for molecular mimicry of heparin binding.. Mol Microbiol.

[10] Choy HA, Kelley MM, Chen TL, Moller AK, Matsunaga J (2007). Physiological osmotic induction of *Leptospira interrogans* adhesion: LigA and LigB bind extracellular matrix proteins and fibrinogen.. Infect Immun.

[11] Lin YP, Chang YF (2007). A domain of the *Leptospira* LigB contributes to high affinity binding of fibronectin.. Biochem Biophys Res Commun.

[12] Lin YP, Chang YF (2008). The C-terminal variable domain of LigB from *Leptospira* mediates binding to fibronectin.. J Vet Sci.

[13] Lin YP, Greenwood A, Nicholson LK, Sharma Y, McDonough SP (2009). Fibronectin binds to and induces conformational change in a disordered region of *Leptospira* immunoglobulin-like protein B.. J Biol Chem.

[14] Lin YP, Greenwood A, Yan W, Nicholson LK, Sharma Y (2009). A novel fibronectin type III module binding motif identified on C-terminus of *Leptospira* immunoglobulin-like protein, LigB.. Biochem Biophys Res Commun.

[15] Lin YP, Lee DW, McDonough SP, Nicholson L, Sharma Y (2009). Repeated domains of *Leptospira* Immunoglobulin-like proteins interact with elastin and tropoealstin.. J Biol Chem.

[16] Lin YP, Raman R, Sharma Y, Chang YF (2008). Calcium binds to Leptospiral immunoglobulin-like protein, LigB and modulates fibronectin binding.. J Biol Chem.

[17] Hauk P, Macedo F, Romero EC, Vasconcellos SA, de Morais ZM (2008). In LipL32, the major leptospiral lipoprotein, the C terminus is the primary immunogenic domain and mediates interaction with collagen IV and plasma fibronectin.. Infect Immun.

[18] Hauk P, Guzzo CR, Ramos HR, Ho PL, Farah CS (2009). Structure and Calcium-Binding Activity of LipL32, the Major Surface Antigen of Pathogenic *Leptospira* sp.. J Mol Biol.

[19] Hoke DE, Egan S, Cullen PA, Adler B (2008). LipL32 is an extracellular matrix-interacting protein of *Leptospira* spp. and *Pseudoalteromonas tunicata*.. Infect Immun.

[20] Vivian JP, Beddoe T, McAlister AD, Wilce MC, Zaker-Tabrizi L (2009). Crystal structure of LipL32, the most abundant surface protein of pathogenic *Leptospira* spp.. J Mol Biol.

[21] Barbosa AS, Abreu PA, Neves FO, Atzingen MV, Watanabe MM (2006). A newly identified leptospiral adhesin mediates attachment to laminin.. Infect Immun.

[22] Stevenson B, Choy HA, Pinne M, Rotondi ML, Miller MC (2007). Leptospira interrogans Endostatin-Like Outer Membrane Proteins Bind Host Fibronectin, Laminin and Regulators of Complement.. PLoS ONE.

[23] Atzingen MV, Barbosa AS, De Brito T, Vasconcellos SA, Morais ZM (2008). Lsa21, a novel leptospiral protein binding adhesive matrix molecules and present during human infection.. BMC Microbiol.

[24] Carvalho E, Barbosa AS, Gomez RM, Cianciarullo AM, Hauk P (2009). Leptospiral TlyC is an extracellular matrix-binding protein and does not present hemolysin activity.. FEBS Lett.

[25] Matsunaga J, Barocchi MA, Croda J, Young TA, Sanchez Y (2003). Pathogenic *Leptospira* species express surface-exposed proteins belonging to the bacterial immunoglobulin superfamily.. Mol Microbiol.

[26] Palaniappan RU, Chang YF, Hassan F, McDonough SP, Pough M (2004). Expression of leptospiral immunoglobulin-like protein by *Leptospira interrogans* and evaluation of its diagnostic potential in a kinetic ELISA.. J Med Microbiol.

[27] Palaniappan RU, Chang YF, Jusuf SS, Artiushin S, Timoney JF (2002). Cloning and molecular characterization of an immunogenic LigA protein of *Leptospira interrogans*.. Infect Immun.

[28] Faisal SM, Khan MA, Nasti TH, Ahmad N, Mohammad O (2003). Antigen entrapped in the escheriosomes leads to the generation of CD4(+) helper and CD8(+) cytotoxic T cell response.. Vaccine.

[29] Faisal SM, Yan W, Chen CS, Palaniappan RU, McDonough SP (2008). Evaluation of protective immunity of *Leptospira* immunoglobulin like protein A (LigA) DNA vaccine against challenge in hamsters.. Vaccine.

[30] Faisal SM, Yan W, McDonough SP, Chang YF (2009). Variable region of *Leptospira* immunoglobulin like protein A (LigAvar) incorporated in Liposomes and PLGA-Microspheres produces a robust immune response correlating to protective immunity against challenge in hamsters.. Vaccine.

[31] Palaniappan RU, McDonough SP, Divers TJ, Chen CS, Pan MJ (2006). Immunoprotection of recombinant leptospiral immunoglobulin-like protein A against *Leptospira interrogans* serovar Pomona infection.. Infect Immun.

[32] Yan W, Faisal SM, McDonough SP, Divers TJ, Barr SC (2009). Immunogenicity and protective efficacy of recombinant *Leptospira* immunoglobulin-like protein B (rLigB) in a hamster challenge model.. Microbes Infect.

[33] Pretorius HT, Nandi PK, Lippoldt RE, Johnson ML, Keen JH (1981). Molecular characterization of human clathrin.. Biochemistry.

[34] Smith AD, Winkler H (1967). Purification and properties of an acidic protein from chromaffin granules of bovine adrenal medulla.. Biochem J.

[35] Tu AT (1982). Raman Spectroscopy in Bology: Principles and Applications.

[36] Meenan NA, Visai L, Valtulina V, Schwarz-Linek U, Norris NC (2007). The tandem beta-zipper model defines high affinity fibronectin-binding repeats within *Staphylococcus aureus* FnBPA.. J Biol Chem.

[37] Patil A, Nakamura H (2006). Disordered domains and high surface charge confer hubs with the ability to interact with multiple proteins in interaction networks.. FEBS Lett.

[38] Kreikemeyer B, Oehmcke S, Nakata M, Hoffrogge R, Podbielski A (2004). *Streptococcus pyogenes* fibronectin-binding protein F2: expression profile, binding characteristics, and impact on eukaryotic cell interactions.. J Biol Chem.

[39] Hammel M, Nemecek D, Keightley JA, Thomas GJ, Geisbrecht BV (2007). The *Staphylococcus aureus* extracellular adherence protein (Eap) adopts an elongated but structured conformation in solution.. Protein Sci.

[40] Ingham KC, Brew S, Vaz D, Sauder DN, McGavin MJ (2004). Interaction of *Staphylococcus aureus* fibronectin-binding protein with fibronectin: affinity, stoichiometry, and modular requirements.. J Biol Chem.

[41] Schwarz-Linek U, Pilka ES, Pickford AR, Kim JH, Hook M (2004). High affinity streptococcal binding to human fibronectin requires specific recognition of sequential F1 modules.. J Biol Chem.

[42] Millard CJ, Ellis IR, Pickford AR, Schor AM, Schor SL (2007). The role of the fibronectin IGD motif in stimulating fibroblast migration.. J Biol Chem.

[43] Vakonakis I, Staunton D, Ellis IR, Sarkies P, Flanagan A (2009). Motogenic sites in human fibronectin are masked by long range interactions.. J Biol Chem.

[44] Callahan B, Nguyen K, Collins A, Valdes K, Caplow M Conservation of structure and protein-protein interactions mediated by the secreted mycobacterial proteins EsxA, EsxB, and EspA.. J Bacteriol.

[45] Laue T, Shah B, Ridgeway T, Pelleitier S, Harding S, Rowe A, Horton J (1992). Computer-aided interpretation of analytical sedimentation data for proteins..

[46] Schuck P (2000). Size-distribution analysis of macromolecules by sedimentation velocity ultracentrifugation and lamm equation modeling.. Biophys J.

[47] van Holde KS (1971). Physical Biochemistry: Prentice-Hall.

[48] Benevides JM, Overman SA, Thomas GJ (2004). Raman spectroscopy of proteins.. Curr Protoc Protein Sci.

[49] Strickland AD, Batt CA (2009). Detection of carbendazim by surface-enhanced Raman scattering using cyclodextrin inclusion complexes on gold nanorods.. Anal Chem.

[50] Litvinovich SV, Strickland DK, Medved LV, Ingham KC (1991). Domain structure and interactions of the type I and type II modules in the gelatin-binding region of fibronectin. All six modules are independently folded.. J Mol Biol.

