# Supplementary figures and images for: The Terminal Immunoglobulin-Like Repeats of LigA and LigB of Leptospira Enhance Their Binding to Gelatin Binding Domain of Fibronectin and Host Cells
Source: PLoS One. 2010 Jun 24;5(6):e11301. doi: 10.1371/journal.pone.0011301 (PMC2892007; doi:10.1371/journal.pone.0011301)

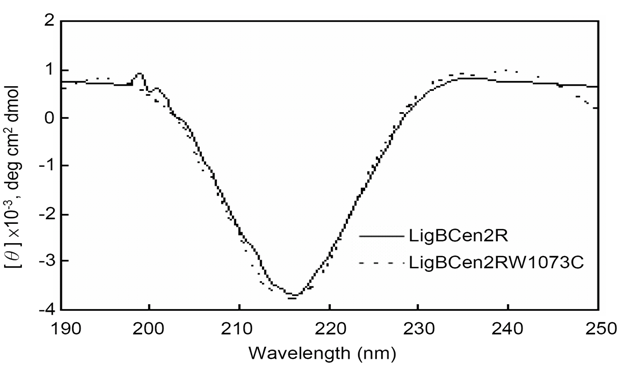

Supplement: Figure S1 — W1073C mutation cannot affect the structure of LigBCen2R. Far-UV CD analysis of LigBCen2R and LigBCen2R. The molar ellipticity, Φ, was measured from 190 to 250 nm for 10 µM of each protein in Tris buffer with 100 µM of calcium chloride. (0.94 MB TIF) [file pone.0011301.s001.tif]

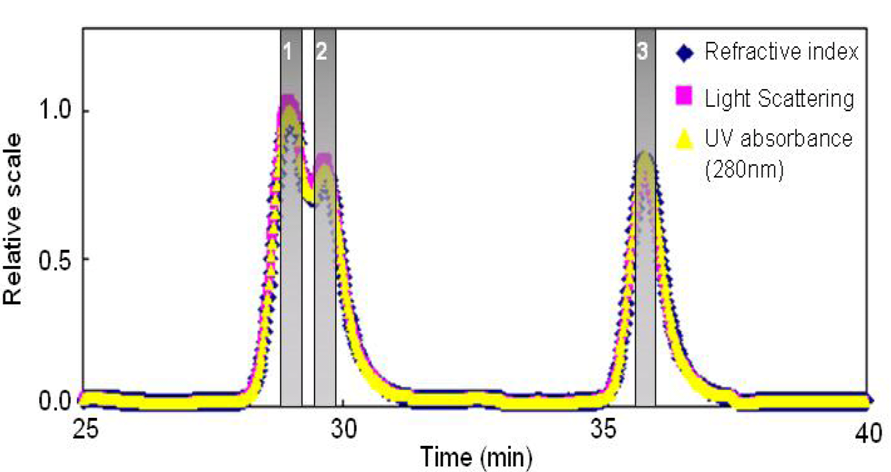

Supplement: Figure S2 — Representative SECMALLS analysis of the molar ratio of LigBCen7'-8-GBD complex. The data traces of SECMALLS from the instrument's three in-line detectors, measuring the refractive index, light scattering, and UV absorbance (280nm), are shown in arbitrary unit. Molecular weight was determined for the major species using the data within the shaded area. Shaded area 1, 2, and 3 indicate LigBCen7'-8-GBD complex, GBD, and LigBCen7'-8, respectively. The result of molar ratio was shown on Table 4. (1.66 MB TIF) [file pone.0011301.s002.tif]
