# Supplementary material for: The Terminal Immunoglobulin-Like Repeats of LigA and LigB of Leptospira Enhance Their Binding to Gelatin Binding Domain of Fibronectin and Host Cells
Source: PLoS One. 2010 Jun 24;5(6):e11301. doi: 10.1371/journal.pone.0011301 (PMC2892007; doi:10.1371/journal.pone.0011301)
Supplement: Table S1 — The sources of clones used in this study. (0.06 MB DOC) [file pone.0011301.s003.doc]

Table S1. The sources of clones used in this study

| Clone | Vector | Source | Tag | Extinction coefficient | Reference |
| --- | --- | --- | --- | --- | --- |
| LigBCon | pGEX4T2 | Residues 1-630 of LigB from *L. interrogans* | GST-tag | 53440 | 31 |
| LigAVar7’-8 | pGEX4T2 | Residues 631-765 of LigA from *L. interrogans* | GST tag | 13490 | This study |
| LigAVar7’-9 | pGEX4T2 | Residues 631-856 of LigB from *L. interrogans* | GST tag | 23140 | This study |
| LigAVar7’-10 | pGEX4T2 | Residues 631-946 of LigB from *L. interrogans* | GST tag | 30110 | This study |
| LigAVar7’-11 | pGEX4T2 | Residues 631-1038 of LigB from *L. interrogans* | GST tag | 38360 | This study |
| LigAVar7’-12 | pGEX4T2 | Residues 631-1140 of LigB from *L. interrogans* | GST tag | 44500 | This study |
| LigAVar7’-13 | pGEX4T2 | Residues 631-1225 of LigB from *L. interrogans* | GST tag | 49620 | 31 |
| LigAVar9 | pQE30 | Residues 756-856 of LigA from *L. interrogans* | Histidine tag | 12210 | This study |
| LigAVar10 | pQE30 | Residues 847-946of LigA from *L. interrogans* | Histidine tag | 6970 | This study |
| LigAVar11 | pQE30 | Residues 938-1038 of LigA from *L. interrogans* | Histidine tag | 9530 | This study |
| LigAVar12 | pQE30 | Residues 1029-1140 of LigA from *L. interrogans* | Histidine tag | 13370 | This study |
| LigAVar13 | pQE30 | Residues 1131-1225 of LigA from *L. interrogans* | Histidine tag | 6970 | This study |
| LigBCen | pQE30 | Resdues 631-1417 of LigB from *L. interrogans* | Histidine tag | 58140 | 12 |
| LigBCen7’-8 | pQE30 | Residues 631-756 of LigB from *L. interrogans* | Histidine tag | 6970 | 15 |
| LigBCen7’-9 | pQE30 | Residues 631-850 of LigB from *L. interrogans* | Histidine tag | 13940 | This study |
| LigBCen7’-10 | pQE30 | Residues 631-941 of LigB from *L. interrogans* | Histidine tag | 22190 | This study |
| LigBCen7’-11 | pQE30 | Residues 631-1033 of LigB from *L. interrogans* | Histidine tag | 31720 | This study |
| LigBCen7’-12 | pQE30 | Residues 631-1124 of LigB from *L. interrogans* | Histidine tag | 39970 | This study |
| LigBCen9 | pQE30 | Residues 755-850 of LigB from *L. interrogans* | Histidine tag | 6970 | 15 |
| LigBCen10 | pQE30 | Residues 846-941 of LigB from *L. interrogans* | Histidine tag | 8250 | 15 |
| LigBCen11 | pQE30 | Residues 936-1028 of LigB from *L. interrogans* | Histidine tag | 9530 | 15 |
| LigBCen2 | pQE30 | Residues 1014-1165 of LigB from *L. interrogans* | Histidine tag | 12090 | 11 |
| LigBCen2R | pGEX4T2 | Residues 1014-1124 of LigB from *L. interrogans* | GST-tag | 9530 | 13 |
| LigBCen2NR | pGEX4T2 | Residues 1120-1165 of LigB from *L. interrogans* | GST-tag | 2560 | 13 |
