# Supplementary material for: The Terminal Immunoglobulin-Like Repeats of LigA and LigB of Leptospira Enhance Their Binding to Gelatin Binding Domain of Fibronectin and Host Cells
Source: PLoS One. 2010 Jun 24;5(6):e11301. doi: 10.1371/journal.pone.0011301 (PMC2892007; doi:10.1371/journal.pone.0011301)
Supplement: Table S2 — Primer Table (0.05 MB DOC) [file pone.0011301.s004.doc]

Table S2. Primer Table

| Primer/Vector | Sequence* |
| --- | --- |
| LigAVar7’-8fp/pGEX4T2** | gcGGATCCCTTACCGTTTCCAAC |
| LigAVar7’-8rp | gcGTCGACATTGAAGTAAGAATT |
| LigAVar7’-9fp/pGEX4T2 | gcGGATCCCTTACCGTTTCCAAC |
| LigAVar7’-9rp | gcGTCGACCTCAATAAGTTCCGC |
| LigAVar7’-10fp/pGEX4T2 | gcGGATCCCTTACCGTTTCCAAC |
| LigAVar7’-10rp | gcGTCGACCGAAACTACTTTAGC |
| LigAVar7’-11fp/pGEX4T2 | gcGGATCCCTTACCGTTTCCAAC |
| LigAVar7’-11rp | gcGTCGACGTAACGAAGAAGCGC |
| LigAVar7’-12fp/pGEX4T2 | gcGGATCCCTTACCGTTTCCAAC |
| LigAVar7’-12rp | gcGTCGACATTTACTATACCACT |
| LigAVar9fp/pQE30*** | gcGGATCCTACCGTTACTCCCGC |
| LigAVar9rp | gcGTCGACCTCAATAAGTTCCGC |
| LigAVar10fp/pQE30 | gcGGATCCTTATCCGTTACCGCA |
| LigAVar10rp | gcGTCGACCGAAACTACTTTAGC |
| LigAVar11fp/pQE30 | gcGGATCCTTCCAAGTTACTCCG |
| LigAVar11rp | gcGTCGACGTAACGAAGAAGCGC |
| LigAVar12fp/pQE30 | gcGGATCCTTGAATGTCACTCCA |
| LigAVar12rp | gcGTCGACATTTACTATACCACT |
| LigAVar13fp/pQE30 | gcGGATCCgttacggttacggaa |
| LigAVar13rp | gcGTCGACTTATGGCTCCGTTTT |
| LigBCen7’-9fp/pQE30 | cgcGGATCCattgctgaaatt |
| LigBCen7’-9rp | cgccCTGCAGaatcggaattgg |
| LigBCen7’-10fp/pQE30 | cgcGGATCCattgctgaaatt |
| LigBCen7’-10rp | cgccCTGCAGaaaatttattttatt |
| LigBCen7’-11fp/pQE30 | cgcGGATCCattgctgaaatt |
| LigBCen7’-11rp | cgccCTGCAGgaccgttatgtc |
| LigBCen7’-12fp/pQE30 | cgGCATGCattgctgaaatt |
| LigBCen7’-12rp | cgAAGCTTgtttactgtgagaat |
| LigBCen2RW1073Cf**** | TCTTCGGTTACATGTTCCAGCTCAAAT |
| LigBCen2RW1073Cr | ATTTGAGCTGGAACATGTAACCGAAGA |

* The restriction enzyme cutting were underlined. ** GE Healthcare, Piscataway, NJ, *** Qiagen Inc., Volencia, CA, **** Primers used for site directed mutagenesis.
